# Decreased Renal Function Is Associated with Elevated CHA2DS2VASC and R2CHADS2 Scores in Non-Valvular Atrial Fibrillation Patients Presenting with Stroke

**DOI:** 10.7759/cureus.1935

**Published:** 2017-12-11

**Authors:** Mohinder Vindhyal, Shravani R Vindhyal, Travis Haneke, Paul M Ndunda, Freidy Eid, K. James Kallail

**Affiliations:** 1 Internal Medicine, University of Kansas School of Medicine - Wichita; 2 Cardiology, Robert J Dole Va Hospital

**Keywords:** non-valvular atrial fibrillation, renal failure, stroke

## Abstract

Introduction

Atrial fibrillation (AF), the most common cardiac arrhythmia, affects approximately 2.3 million patients in the United States, costing around $26 billion. Atrial fibrillation is associated with a two- to seven-fold increased risk of stroke, one of the most serious complications. Chronic kidney disease affects approximately 13% of the US population and has been associated with higher rates of AF than the general population. In patients with chronic kidney disease (CKD), the risk of stroke increases as the glomerular filtration rate (GFR) decreases, especially in CKD stages three and four.

Several risks stratification scores such as CHADS2 (congestive heart failure, hypertension, age, diabetes mellitus, stroke), CHA2DS2VASc (congestive heart failure, hypertension, age, diabetes mellitus, stroke, vascular disease, age, sex), and R2CHADS2 (renal failure, congestive heart failure, age, diabetes, stroke) scores are used for stroke risk assessment in patients with non-valvular atrial fibrillation (NVAF). This study investigates the association between renal functions and risk stratification scoring systems in patients with non-valvular AF presenting with stroke.

Methods

Using the convenience sampling method, 171 subjects were selected from the eligible population (n = 386). A Pearson product-moment correlation coefficient was calculated to determine the association between the GFR and each of the CHA2DS2VASc and R2CHADS2 scores. In addition, a Pearson product-moment correlation coefficient was calculated to determine the association between the CHA2DS2VASc and R2CHADS2 scores.

Results

The selected population represented 44.3% of the eligible subjects. Of these, 88% were Caucasian, 60% were female, and the mean age was 78 years. The mean CHA2DS2VASc score was six (range 2-9). The mean eGFR was 69.77 (range 6-108). Both the mode and the median CHA2DS2VASc score was four (range 2-8). A weak, but significant, negative correlation was found between renal function (eGFR) and the CHA2DS2VASc score (r = -0.263; p = 0.0005). There was a stronger negative correlation between the eGFR and R2CHADS2score (r = -0.70; p < 0.00001). The CHA2DS2VASc and R2CHADS2scoring schemes were significantly and positively correlated (r = 0.627; p < 0.00001).

Discussion

In NVAF patients presenting with stroke, renal failure is associated with higher CHA2DS2VASc and R2CHADS2 scores. One must consider renal failure (end-stage or non-end stage renal failure) as an additional potential risk factor for stroke when recommending anticoagulation in non-valvular atrial fibrillation.

## Introduction

Atrial fibrillation (AF) is the most common cardiac arrhythmia [1] affecting approximately 2.3 million Americans. The number is expected to increase to 5.6 million by 2050 [2]. Every year, AF costs around $26 billion, causing a substantial burden to the healthcare system [3]. Atrial fibrillation is associated with a two- to seven-fold increased risk of stroke, one of the most serious complications [4]. Many risk factors contributing to AF have been identified; among them, the non-modifiable factors are age and gender. Other factors include hypertension, valvular heart disease, congenital heart disease, heart failure, coronary artery disease, obesity, diabetes mellitus, smoking, alcohol consumption, thyroid disease, and chronic kidney disease [5]. Of the many risk factors for AF, chronic kidney disease (CKD) is a potentially modifiable risk factor. Chronic kidney disease affects approximately 13% of the US population [6] and has been associated with higher rates of AF than the general population [7]. In patients with CKD, the risk of stroke increases as the glomerular filtration rate (GFR) decreases, especially in CKD stages three and four [8].

Both AF and CKD increase the risk of thromboembolism. In the US, approximately 800,000 new strokes are reported yearly [9], which can have severe and devastating effects. Because of the potential for severe sequela from stroke, several risk stratification scores have been developed to aid in identifying patients requiring oral anticoagulation (OAC) to prevent future thromboembolic events [10]. The most popular scores are the CHADS2 (congestive heart failure, hypertension, age, diabetes mellitus, stroke), CHA2DS2VASc (congestive heart failure, hypertension, age, diabetes mellitus, stroke, vascular disease, age, sex), and R2CHADS2 (renal failure, congestive heart failure, age, diabetes, stroke) scores. While each has a unique scoring system, they are alike in that they score one point for congestive heart failure, hypertension, and diabetes, and two points for previous episodes of stroke or thromboembolism. In the initial CHADS2 score, age greater than or equal to 75 years is given one point. However, in the updated CHA2DS2VASc score, one point is given for ages 65-74 years and two points for ages greater than or equal to 75 years. The latter scoring system also adds one point for vascular disease and the female sex. Neither of these risk stratification scores includes renal failure as a predictive prognostic factor, and both are endorsed by American College of Cardiology/American Heart Association (ACC/AHA). R2CHADS2 is another scoring system which includes two points for renal failure (stage 2 or greater) and stroke and one point for congestive heart failure, hypertension, and diabetes.

Patients with CKD and AF share many of the same risk factors for stroke; it is unclear if renal failure is itself an independent risk factor. Previous studies have attempted to evaluate the addition of renal failure as an independent variable to current risk stratification schemes [11]. However, these studies have suggested conflicting results as to whether or not renal failure serves as an independent risk factor in predicting stroke.

This study aims to evaluate renal failure as a risk factor for thromboembolic events in patients previously diagnosed with nonvalvular atrial fibrillation (NVAF). Improvements in stroke risk stratification scores can further aid in properly determining which patients should receive anticoagulation. The information could be useful in reducing the number of adverse effects, such as hemorrhage or stroke, from incorrect use of therapies.

## Materials and methods

Research study design

This is a retrospective chart review.

Participants

Patients were selected from a Kansas hospital database from 2012 through 2014. To be included, patients older or of 18 years of age must have presented to the hospital with a stroke and a prior diagnosis of atrial fibrillation. There was no risk to patients.

Instruments

Patients meeting all inclusion criteria had the following parts of their charts reviewed: history and physical, lab data, admission echocardiogram, and discharge documents only from the current admission. The study variables included: medical record number, age, race, ejection fraction, creatinine, and history of hypertension, diabetes, vascular disease, congestive heart failure, and prior stroke. This study defined heart failure as either any diastolic heart failure or a systolic ejection fraction less than 40% as reported on an echocardiogram performed during the current hospital admission. Creatinine was collected to assess renal function, and the lowest creatinine value during the current hospitalization was selected for use as the best estimate of the patient’s baseline value. Data on race was also collected to determine the estimated GFR using the CKD-EPI creatinine equation [12]. Calculation of the CHA2DS2VASc risk stratification score included assigning one point for the following: congestive heart failure, hypertension, age 65-74 years, diabetes mellitus, vascular disease (history of myocardial infarction, peripheral vascular disease, or aortic atherosclerosis), and female sex.  Age greater than or equal to 75 years was assigned two points as was a prior history of stroke. Scoring for CHADS2 was one point for congestive heart failure, hypertension, age greater than or equal to 75 years, and diabetes mellitus, and two points were assigned for stroke. To calculate the R2CHADS2 score, the CHADS2 scoring system was used with an additional two points assigned for renal failure, which was defined as a calculated GFR less than 60 from the CDK-EPI equation.

Procedures

This study was approved by the institutional review boards at the University of Kansas School of Medicine-Wichita and Via Christi Hospitals of Wichita, KS. A convenience sample of 171 subjects from all eligible patients (n = 386) meeting the study criteria was assigned by the study director for review. The charts were reviewed by investigators, and the aforementioned data was collected and entered into a data sheet stored on password-secured computers. The data sheets were compiled into one data sheet for analysis. A convenience sample was used because as it was more a pilot study to obtain basic data and trends regarding the association. The demographics of the patient population can be seen in Table 1.

**Table 1:** Clinical characteristics of patients GFR: glomerular filtration rate CKD - EPI: chronic kidney disease epidemiology collaboration

	Number of Subjects (%)	Mean GFR (CKD - EPI)	Number of Subjects with Renal Failure (%)
ALL PATIENTS	171 (100)	69.77	49 (28.7)
SEX			
Male	69	70.90	19 (11.1)
Female	102	69.01	30 (17.5)
AGE			
<65	23 (13.5)		
65-75	37 (21.6)		
>75	111 (64.9)		
COMORBIDITIES			
Heart failure	54 (31.6)		
Hypertension	143 (21.6)		
Diabetes	57 (33.3)		
Vascular disease	79 (46.2)		

Analysis

A Pearson product-moment correlation coefficient was calculated to determine the association between the GFR and each of the CHA2DS2-VASc and R2CHADS2 scores. In addition, a Pearson product-moment correlation coefficient was calculated to determine the association between the CHA2DS2-VASc and R2CHADS2 scores. Summary statistics were calculated for each dependent variable.

## Results

**Figure 1:**
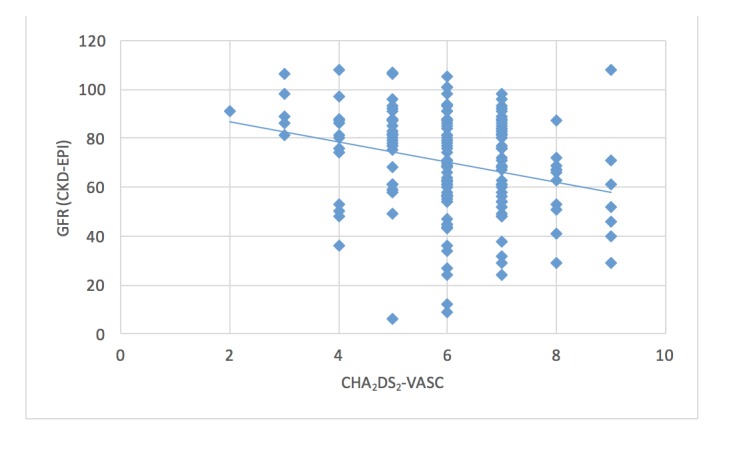
Correlation between CHA2DS2-VASC and GFR (r = -0.263; p = 0.0005) GFR: glomerular filtration rate CHA2DS2-VASC: congestive heart failure, hypertension, age, diabetes mellitus, stroke, vascular disease, age, sex

**Figure 2:**
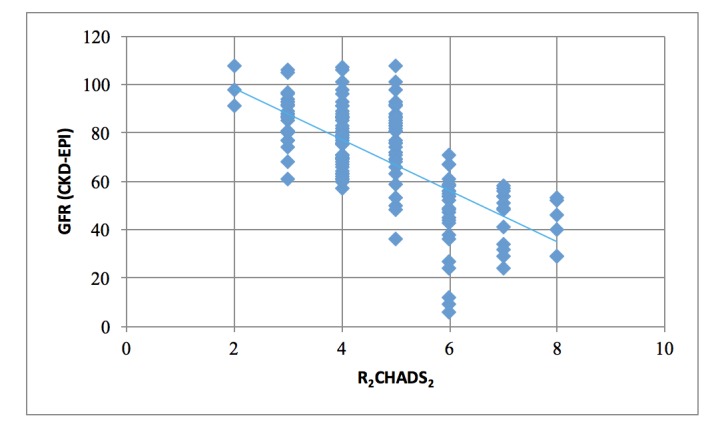
Correlation between R2CHADS2 and GFR (r = -0.70; p R2CHADS2: renal failure, congestive heart failure, age, diabetes, stroke GFR: glomerular filtration rate

Of the total, 386 subjects met the eligibility criteria. A convenience sample of 171 subjects was selected, thus representing 44.3% of all eligible subjects. Among those sampled, 88% were Caucasian and 60% were female. The mean age was 78 years. The mean CHA2D2VASc score was six (range 2-9). The mean eGFR was 69.8 (range 6-108). Both the mode and the median R2CHA2D2VASc score was four (range 2-8). A weak, but significant, negative correlation was found between renal function (eGFR) and the CHA2DS2-VASc score (Figure 1; r = -0.263; p = 0.0005). There was a stronger negative correlation between the eGFR and R2CHADS2 scores (Figure 2; r = -0.70; p < 0.00001). The CHA2DS2-VASc and R2CHADS2 scoring schemes were significantly and positively correlated (r = 0.627; p < 0.00001).

## Discussion

This study highlights that in patients with NVAF, renal failure is associated with increased risk of stroke. At first glance, the negative correlation may be a bit confusing. As eGFR decreases, the CHA2DS2VASc score increases. Because a higher CHA2DS2VASc is associated with a higher risk of stroke, this means that as eGFR decreases, the yearly risk of stroke increases. The correlation between the eGFR and CHA2DS2VASc score also suggests the possibility that the risk of stroke progressively increases the further eGFR declines; however, the exact extent of this association is not well established. Alternatively, it is possible that no true relationship exists between these two variables but, rather, it is merely due to the sharing of the same risk factors. It is of note that this study does not compare renal failure with other risk factors in stroke risk stratification schemes in their ability to predict yearly stroke risk.

Another, yet stronger, negative correlation was found between eGFR and the R2CHADS2 score. Again, this suggests that renal failure, in this particular scoring system, is also associated with a higher risk of stroke. It is not surprising that this correlation exists and is stronger since the R2CHADS2 score includes renal failure as one of its components.  

Additionally, there is a significant positive correlation between the CHA2DS2VASc and R2CHADS2 scores. This should not be a surprise as the two scoring systems share many of the same scoring components and are similar in their weighting of each component. Because they are correlated, this suggests the validity of using the R2CHADS2 score to predict the risk of stroke. However, the addition of renal failure may not change clinical decisions or outcomes since the CHA2DS2VASc has already been validated and is currently being used.

These findings complement prior research highlighting a correlation between renal failure and stroke risk in patients with NVAF [13]. It also supports research suggesting the potential benefit of adding renal failure to current stroke risk prediction schemes [14]. While the risks and benefits of OAC use should always be reviewed on an individual basis, the results of this study support anticoagulation in CKD patients with NVAF in order to prevent future thromboembolic events [15].

Due to the high morbidity and mortality associated with thromboembolism, it is important to attempt to control modifiable risk factors, such as renal function. While many of the risk factors for CKD, AF, and stroke are the same, renal failure may prove to be a separate independent risk factor for thromboembolism. It would be reasonable to consider the addition of renal failure to stroke risk prediction scores to better assess the risks and benefits of OAC in patients with kidney disease.  

There are several limitations to this study. First, the generalizability of the results of this study is limited, as this was a small sample collected from a single Midwest hospital over a limited two-year period. Despite the small sample size, it provides a basis for further research in evaluating the role of renal function in patients with NVAF and risk of stroke. Secondly, this study compares eGFR to both the CHA2DS2VASc and R2CHADS2 scores. These scoring schemes vary in the number of points assigned for each risk factor. Most notably, scoring for age varies widely between these two schemes. Because of this difference, it is difficult to compare these head to head. While one could argue the need for a comparable R2CHA2DS2VASc, the decision to use the R2CHADS2 score derives from its validation in predicting stroke risk [16]. 

Another limitation was the decision to use the best creatinine value during hospitalization. While this was used as the best estimate of the patient’s normal baseline creatinine, it may not accurately reflect kidney function as other confounding variables may have adversely affected it during hospitalization. Thus, eGFR calculations may have been altered, although the decision to use the GFR-CKD/EPI equation over other estimated eGFR calculations, such as the Modification of Diet in Renal Disease (MDRD) equation, should improve accuracy, specifically with higher renal function [17]. A larger sample size may help to eliminate some of the potential variability in creatinine values and better aid in accurately evaluating the correlation between renal failure and stroke risk.

## Conclusions

In patients with NVAF presenting with stroke, renal failure is associated with higher CHA2DS2VASc and R2CHADS2 scores. One must consider renal failure (end-stage or non-end stage renal failure) as an additional potential risk factor for stroke when recommending anticoagulation in non-valvular atrial fibrillation. There is a further need for a randomized control trial to consider renal failure as an independent risk factor for stroke in non-valvular atrial fibrillation.
